# Functional connectivity and cognitive impairment in migraine with and without aura

**DOI:** 10.1186/s10194-017-0782-6

**Published:** 2017-07-20

**Authors:** Viviana Lo Buono, Lilla Bonanno, Francesco Corallo, Laura Rosa Pisani, Riccardo Lo Presti, Rosario Grugno, Giuseppe Di Lorenzo, Placido Bramanti, Silvia Marino

**Affiliations:** 1 IRCCS Centro Neurolesi “Bonino-Pulejo”, S.S. 113 Via Palermo, C.da Casazza, 98124 Messina, Italy; 2 Department of Biomedical and Dental Sciences and Morphological and Functional Imaging, University of Messina, Messina, Italy

**Keywords:** Migraine, Cognitive functions, Functional connectivity, default mode network

## Abstract

**Background:**

Several fMRI studies in migraine assessed resting state functional connectivity in different networks suggesting that this neurological condition was associated with brain functional alteration. The aim of present study was to explore the association between cognitive functions and cerebral functional connectivity, in default mode network, in migraine patients without and with aura, during interictal episodic attack.

**Methods:**

Twenty-eight migraine patients (14 without and 14 with aura) and 14 matched normal controls, were consecutively recruited. A battery of standardized neuropsychological test was administered to evaluate cognitive functions and all subjects underwent a resting state with high field fMRI examination.

**Results:**

Migraine patients did not show abnormalities in neuropsychological evaluation, while, we found a specific alteration in cortical network, if we compared migraine with and without aura. We observed, in migraine with aura, an increased connectivity in left angular gyrus, left supramarginal gyrus, right precentral gyrus, right postcentral gyrus, right insular cortex.

**Conclusion:**

Our findings showed in migraine patients an alteration in functional connectivity architecture. We think that our results could be useful to better understand migraine pathogenesis.

## Background

Migraine is a common episodic neurological disorder with a complex physiopathology. It is characterized by typical unilateral, often severe, pain throbbing with associated features such as hypersensitivity to multiple stimuli, including visual (photophobia), auditory (phonophobia), and sensory (cutaneous allodynia) stimuli during migraine attacks [1]. Indeed, about one third of patients had experience of aura associated to visual, motor, or somatosensory symptoms during attacks [2, 3].

Migraine is a very common and debilitating disease that causes significant limitations in daily life with effects on emotional-behavioral and relational aspects [4]. Neuropsychological studies suggests that migraine affect also cognitive functions during attacks and interictal periods [5], even though it is unclear the association between cognitive dysfunctions and migraine. Migraineurs could present executive dysfunction which presumably reflects frontal lobe abnormalities [6], or alteration in memory areas. However, while several authors reported significant lower performances in migraine patients, others did not confirm these findings. In other cases authorsdescribed the presence of cognitive deficit only after a long disease duration [7, 8].

Several fMRI studies in migraine assessed resting state functional connectivity in various networks suggesting an association with cortical functional alteration [9]. In particular, some authors reported increased connectivity in specifics cerebral areas, such as right rostral anterior cingulate cortex, prefrontal cortex, orbitofrontal cortex and supplementary motor area [10]. This altered connectivity could indicate intrinsic pathophysiological changes in migraine, even if only a very few studies explored the different functional connectivity in migraine with (MA) and without aura (MO) [11].

The aim of present study was to explore the association between cognitive functions and cerebral functional connectivity (FC) between MO and MA, during interictal episodic attack.

## Methods

Twenty-eight migraine patients (14 without aura and 14 with aura) and 14 sex and age matched health controls (HC), were enrolled. Aura included temporary visual or sensory disturbances nausea, and sensitivity to light and sound. The patients were recruited from migraine ambulatory. The diagnosis of definite MA or MO was performed by two neurologist, specialist in headache disorders, blinded to MRI and neuropsychological findings, according to International Headache Society criteria [12] (Headache Classification Committee of the International Headache, 2013).

Control subjects were volunteers recruited from local communities, with no history of neurological diseases. They did not suffer from migraine or headache and were free from medication intake. The study protocol was approved by the Local Ethics Committee according to Declaration of Helsinki. All patients gave written consent to study. All information related to migraine was collected by interviews and examination of medical records. All patients had a clinic diagnosis for at least 10 years. We excluded patients with: 1) other types of headache; 2) vascular disease or trauma; 3) history of major psychiatric disorders; 4) presence of metabolic disorders; 5) other neurological conditions.

Demographic and clinical characteristics were also collected (Table 1). The type of medication, during attack, in patient included: simple analgesics (18/24), simple triptens (4/24), and combination analgesics (6/24).

**Table 1:** Socio-demographic characteristics of patients with aura (*n* = 14) without aura (*n* = 14) and controls (*n* = 14)

	Aura (Mean ± SD)	No Aura (Mean ± SD)	HC (Mean ± SD)
Age	41.28 ± 13.44	40.75 ± 11.82	41.75 ± 12.82
Years of education	15.8 ± 3.2	16.7 ± 4.2	16.2 ± 4.1
Disease duration	10.9 ± 3.7	12.3 ± 5.8	
Attack frequency/month (n)	5.05 ± 2.31	6.07 ± 2.81	
Single-Attack duration (hours)	3.58 ± 2.27	4.21 ± 2.99	
Days to next migraine attack after examination			

Legend: *SD* standard deviation

A battery of standardized neuropsychological test to evaluate cognitive functions, was administered by two psychologists, blinded to patients/controls status, diagnosis and MRI findings. Processing speed was assessed using the Trail Making Test, Part A (TMT-A), [13]. Attentional set-shifting was measured using the Trail Making Test, Part B (TMT-B). Memory was assessed using the Rey Auditory Verbal Learning Test (RAVLT) [14]. Language was assessed with semantic and phonemic verbal fluency test [15]. Wisconsin Card Sorting test (WCST) was used for executive function and cognitive flexibility. Finally, Hamilton Rating Scale for depression (HAM-D) and Hamilton Rating Scale for anxiety (HAM-A) were used to asses anxiety and depressive symptoms [16, 17].

All patient underwent to a MRI examination with a scanner operating at 3.0 T (Achieva, Philips Healthcare, Best, The Netherlands), by using a 32-channel SENSE head coil. MRI scans were performed in the interictal stage at least 3 days after migraine attack. For each subject, T1 [TR = 8 ms, TE = 4 ms, slice thickness/gap = 1/0 mm, number of slices = 173, field of view 240 mm], T2-weighted [TR = 3.0 s, TE = 80 ms, slice thickness/gap = 3.0/0.3 mm, number of slices = 30, field of view 230 mm] were acquired. The scan parameters of the resting-state functional magnetic resonance imaging (fMRI) scan were as follows: TR = 3.0 s; TE = 35 msec; flip angle = 90°; and voxel size 1.9 · 1.9 · 4.0 mm, scan duration 10 min. During the resting-state scan, participants were instructed to lie still with their eyes closed and not to fall asleep.

Neuropsychological testing and MRI scanning were performed on same day.

### Resting state analysis

fMRI-analysis was performed with FSL (FMRIB’s Software Library, www.fmrib.ox.ac.uk/fsl). The following pre-processing procedure was applied: employing different modules of the FSL-software package. The preprocessing of the resting-state data consisted of motion correction (MCFLIRT) [18], brain extraction [19], spatial smoothing using a Gaussian kernel with a full width at a half maximum of 8 mm. After preprocessing, the functional images were registered to the corresponding high-resolution echo planar images, (co-registered to T1-weighted images,) which were registered to the 2 mm isotropic MNI-152 standard space image [18]. These registration parameters were combined to obtain registration matrix from native (fMRI) space to MNI space and its inverse (from MNI space to native space). Independent component analysis (ICA) was carried out using MELODIC toolbox implementing probabilistic independent component analysis (PICA) [20]. Variance normalization was used and IC maps were thresholded using an alternative hypothesis test based on fitting a Gaussian/gamma mixture model to distribution of voxel intensities within spatial maps and controlling the local false-discovery rate at *p* < 0.5 [20]. The selection of clusters of interest obtained of MELODIC analysis implied the presence of anatomically relevant areas in each group component map that reproduced the layouts of the main physiological resting state network jointly and consistently across subjects. The artefact components were removed manually from analysis and for all groups we considered IC of the DMN, one of the main networks that are consistently identified when an individual is at wakeful rest and not performing an attention-demanding task. This network includes the precuneus, posterior cingulate cortex (PCC), medial prefrontal cortex, medial temporal lobe and angular gyrus. For inter group analysis was carried out using dual regression (FSL technique) that allows for voxel-wise comparisons of resting-state [21, 22]. This allow, a) to separate fMRI data sets using the group-ICA spatial maps in a linear model fit against, resulting in matrices (time-course matrices) describing the temporal dynamics for each component and subject, and b) estimate subject-specific spatial maps using these time-course matrices. The dual regression analysis was performed with variance normalization because reflects differences in both activity and spatial spread of the network. As a statistical analysis the different component maps are collected across subjects into single 4D files and tested voxel-wise for statistically significant differences between the groups using FSL randomize non parametric permutation testing, with 5000 permutations, using a threshold-free cluster enhanced (TFCE) technique to control for multiple comparisons [23] and corrected for multiple comparisons (across space) within the permutation framework. Age and gender also included in this analysis as nuisance variable. The Harvard-Oxford Cortical structural atlas were used to identify the anatomical characteristics of the resulting PICA maps. Fslstats and fslmaths tools were used to calculate the number of non-zero voxels in the selected difference maps, and their t-score values.

## Results

### Demographic characteristics

Inter group analysis by U Mann Whitney test no highlighted differences between characteristics and clinical scores of patients (Table 1). There were no differences between MA and MO patients in age, (*p* = 0.84), education (*p* = 0.35) and disease duration (*p* = 0.27). Both groups did not show abnormalities in neuropsychological evaluation (Table 2).

**Table 2:** Cognitive performances of the migraine patients

Test	Aura	No Aura	Controls groups	Cut-off
Attention				
Attentive Matrix	44.60 ± 4.80	45.51 ± 6.91	43.35 ± 7.87	30
Language				
Fluency Phonemic	32.08 ± 11.72	35.35 ± 10.9	30.85 ± 6.63	17
Fluency Semantic	36.25 ± 6.64	36.28 ± 5.86	37.42 ± 5.74	25
Memory				
RAVLT (Immediate recall)	40.86 ± 25.01	36.28 ± 5.86	38.17 ± 4.59	28.53
RAVLT (Delayed recall)	8.2 ± 2.45	9.23 ± 3.10	6.85 ± 1.65	4.69
Executive Functions				
Trial Making Test-A	42.62 ± 25.01	48.73 ± 55.20	55.28 ± 15.52	93
Trial Making Test-B	123.35 ± 56.28	155.14 ± 70.16	126.64 ± 30.49	282

### Resting state

#### MA vs MO

**Fig. 1:**
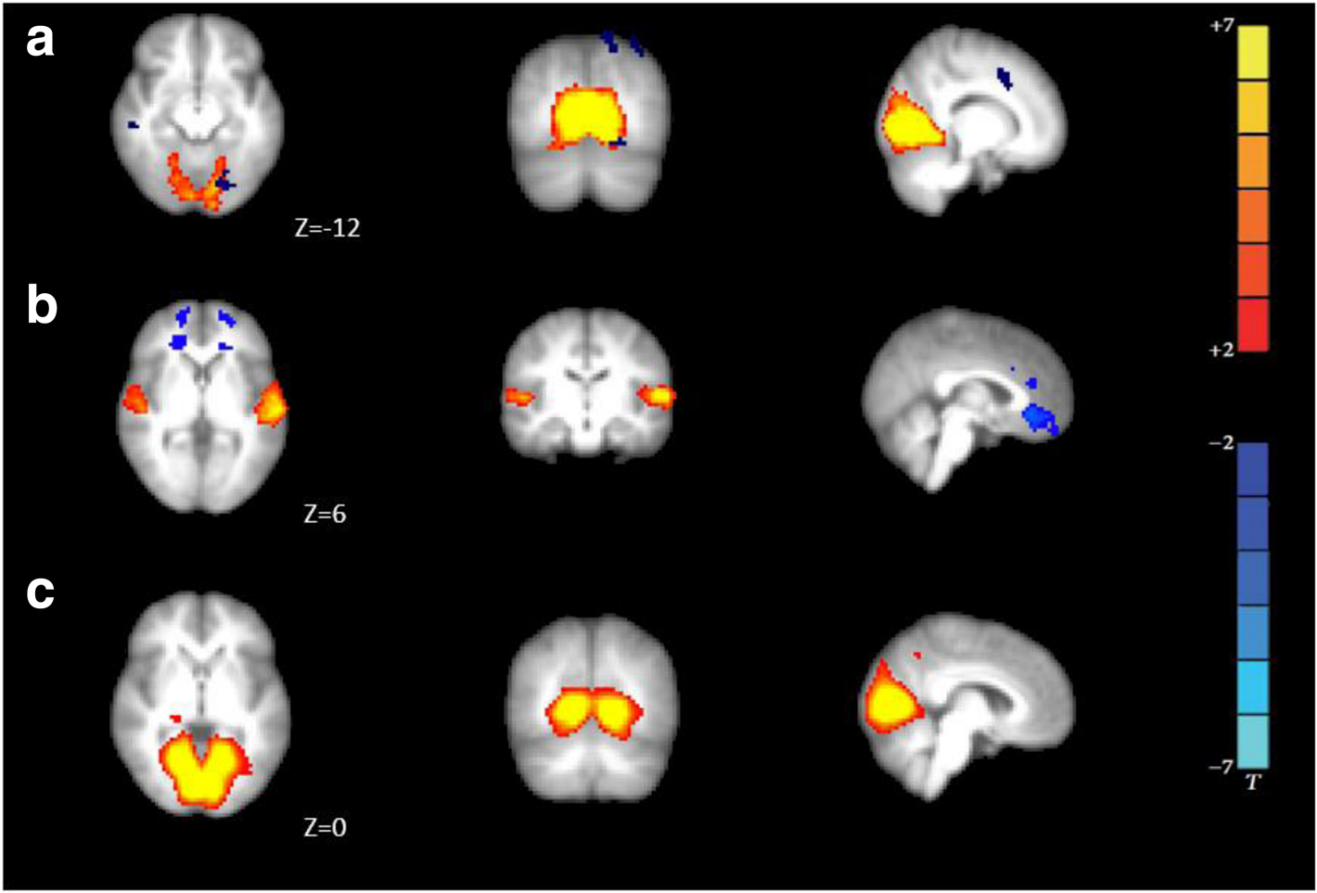
*Functional connectivity average DMN of groups:* **a.** MA > MO group; **b.** MA > HC; **c.** MO > HC group*.* MA patients showed increased functional connectivity compared MO (*blue* areas, *p* values are color coded from 0.05 FWE corrected (*dark blue*) to <0.0001 FWE corrected (*light blue*), full list of structures in Table 2). Axial images are overlaid on transverse slices of MNI-152 standard anatomical image. The left side of the brain corresponds to the right hemisphere and vice versa. Z-coordinates of each slice in the MNI-152 standard space are given

MA group showed increased functional connectivity if compared to MO group (blue area, *p* values are color coded from 0.05 FWE corrected (dark blue) to <0.0001 FWE corrected (light blue). Increased in functional connectivity was found in left angular gyrus, left supramarginal gyrus, right precentral gyrus, right postcentral gyrus, right insular cortex (Fig. 1a, full list of structures are showed in Table 3). No significant voxels for MA < MO were found.

**Table 3:** Increased functional connectivity in MA compared with MO

Brain Structure	Peak voxel coordinates (MNI)			Peak T-score
	x	y	z	
Right Central Opercular Cortex	48	-6	6	3.89
Right Insular cortex	42	−9	6	4.97
Right first and second Heschl’s Gyrus	45	−12	6	4.12
Left Central Opercular Cortex	−45	−9	6	3.17
Left first and second Heschl’s Gyrus	−51	−15	6	3.75
Left Superior Temporal gyrus	−69	−27	6	3.41
Right Lingual gyrus	18	−66	−12	4.61
Right Occipital fusiform gyrus	18	−75	−12	5.48
Left occipital pole	−12	−93	−12	6.60
Left Lingual gyrus	−12	−84	−12	6.82

Harvard-Oxford Cortical structural atlas

For each peak voxel x-, y-, and z-coordinates in the MNI − 152 standard space image are given

#### MA vs HC

Patients showed increased functional connectivity (blue area, *p* values are color coded from 0.05 FWE corrected (dark blue) to <0.0001 FWE corrected (light blue)) in bilateral frontal pole, right paracingulate gyrus, in right first and second Heschl’s gyrus, planum temporale, left in first and second Heschl’s gyrus, planum temporale and superior temporal gyrus (Fig. 1b, full list of structures in Table 4). No significant voxels for MA < HC were found.

**Table 4:** Increased functional connectivity in MA compared with HC group

Brain Structure	Peak voxel coordinates (MNI)			Peak T-score
	x	y	z	
Right Heschi’s	54	-15	6	4.20
Right Planum temporale	54	−21	6	3.97
Left Heschi’s gyrus	−54	−15	6	3.80
Left Planum temporale	−57	−21	6	3.51
Left Superior temporal gyrus	−57	−33	6	3.75

Harvard-Oxford Cortical structural atlas

For each peak voxel x-, y-, and z-coordinates in the MNI-152 standard space image are given

#### MO vs HC

Cerebral regions showed increased functional connectivity in the DMN included right lingual gyrus, occipital fusiform gyrus, occipital pole and cingulate gyrus and, in the left side, increase connectivity in lingual gyrus, occipital fusiform gyrus, occipital pole and cingulate gyrus (Fig. 1c, full list of structures in Table 5) in both groups. No significant voxels for MO < HC were found.

**Table 5:** Increased functional connectivity in MO compared with HC group

Brain Structure	Peak voxel coordinates (MNI)			Peak T-score
	x	y	z	
Right Lingual gyrus	18	−54	0	3.54
Right Occipital fusiform gyrus	21	−75	0	3.21
Right Occipital pole	9	−93	0	4.85
Right Cingulate gyrus	18	−45	0	2.8
Left Lingual gyrus	−18	−54	0	4.0
Left Occipital fusiform gyrus	−21	−75	0	3.1
Left Occipital pole	−9	−93	0	4.57
Left Cingulate gyrus	−12	−45	0	3.5

Harvard-Oxford Cortical structural atlas

For each peak voxel x-, y-, and z-coordinates in the MNI-152 standard space image are given

## Discussion

Recently, several studies investigated the activity of resting state network in migraine and showed alterations in brain functional reorganization. Altered functional connectivity was found in cognitive cerebral networks, such as executive control network, default mode network, visual network. It seem to be associated to disease duration, gender, and migraine chronicity [24–26]. The DMN is a cerebral network related to different regions with relatively greater activity during rest-state than during active conditions [27, 28]. It refers to an interconnected group of brain structures that are hypothesized to be part of a functional system. Although the exact functional role of DMN is not completely know, it is thought to be involved in several cognitive processes, such as memory, problem solving and planning [2, 29]. In DMN, there are heteromodal association areas, which have a high number of connections with brain regions involved in integration processes, including pain matrics. In chronic pain DMN is altered [30], and this is possibly due to the increase of baseline activity of other cognitive, salience, or sensorimotor networks. Over time, chronic pain becomes an intrinsic brain activity occurring even in the absence of explicit brain input or output: thus, the alterations in patient’s brain at “rest” could be considered as a different or altered DMN organization [31]. In our study we identified specific alterations, during resting state examination, in cortical DMN if we compared MA, MO and HC. Our findings showed an increase of functional connectivity, in MA, in frontal and parietal lobes, in particular in angular, supramarginal gyrus, somatosensory association cortex, postcentral gyrus and primary somatosensory cortex. Since pain is inherently salient it is rational to speculate that the intrinsic connectivity in this network may be changed in chronic pain patients, like migraine subjects. In addition, in MA patients, we found an altered connectivity in insular cortex. It is know that insula is involved in triggering of pain matrix network and in the subjective pain experience [32]. It is also implicated in cognitive, affective, and regulatory functions, including interoceptive awareness, emotional responses, empathic and attentional processes [33]. The insula seems to be a cortical hub, to process complex sensory and emotional aspects in the migraine condition [34], through connections in frontal, temporal and parietal cortex, basal ganglia, thalamus and limbic structures. It is important to understand if functional connectivity abnormalities in this network could be correlated to minimal impairments in neuropsychological performances, such as processing speed, verbal memory, as reported in migraine in interictal attack period. In fact, although MA showed a cognitive performance lower than MO in executive functions, we did not find a significant impairment in two groups. In other word, in our patients, connectivity altered in DMN dwas not associate to neuropsychological variables and cognitive performances.

Moreover, we found in MA a greater cortical hyperexcitability than MO: resting-state abnormal activity could play a key role in the pathogenesis knowledge of migraine attacks with aura [35]. In particular, alterations of the DMN functional connectivity in migraine may lead to changes in pain modulating network, which could be considered as a neuroimaging biomarkers for disease pathophysiology.

## Conclusions

The importance of various frequencies of BOLD fluctuations is not yet known, even if recently few studies started to explore this feature, especially in pain conditions. Brain dysfunction affecting intrinsic connectivity in migraine, possibly reflecting the impact of long lasting and constant pain on brain function.

Although our study was limited to a small sample size, our results confirmed that brain functional connectivity in migraine patients showed an alteration of DMN connectivity, suggesting that pain has a widespread impact on brain function, since modify the complex brain networks and beyond pain perception. Although migraine is one of the most investigated neurologic disorders, specific neuroimaging biomarker for its pathophysiology has not been found.Altered intrinsic functional connectivity architecture was identified in migraine patients and our finding could provide a new perspective to understand the pathogenesis of MA and MO migraine, in order to find a more appropriate therapeutic management.
